# Participatory development and proof-of-concept of a dyadic-based, AI-driven, just-in-time adaptive intervention mechanism for preventing anxiety and depressive disorders via app: study protocol for a feasibility study

**DOI:** 10.3389/fpubh.2025.1626428

**Published:** 2025-07-29

**Authors:** Anna-Carlotta Zarski, Theresa Sextl-Plötz, Juliane Schmidt-Hantke, Natalie Sarah Hess, Claudia Buntrock

**Affiliations:** ^1^Division of eHealth in Clinical Psychology, Department of Clinical Psychology, Philipps-Universität Marburg, Marburg, Germany; ^2^Medical Faculty, Institute of Social Medicine and Health Systems Research, Otto-von-Guericke University Magdeburg, Magdeburg, Germany

**Keywords:** AI driven JITAI, mental health app, prevention, mental health, mobile health, internet and mobile intervention, digital mental health, ecological momentary assessment

## Abstract

**Background:**

The growing prevalence of anxiety and depressive disorders highlights the need for transdiagnostic prevention through innovative interventions that outperform existing applications. By delivering personalized, context-sensitive support in moments of need, Just-In-Time-Adaptive-Interventions (JITAIs) have the potential to boost intervention relevance, engagement, and adherence.

**Aims:**

The study aims to (1) develop a transdiagnostic dyadic, AI-based JITAI (DyAI-JITAI) app through a participatory process, and (2) evaluate its feasibility in terms of acceptability, usability, and preliminary intervention effects.

**Methods:**

Stage I followed a participatory development process using focus groups, think-aloud tests, and qualitative interviews to explore expectations, ideas, and needs for a preventive DyAI-JITAI app among *N* = 28 target users with lived experience and potential users without clinical anxiety or depressive symptoms. Stage II involves a randomized-controlled proof-of-concept study (*N* = 60) to test the DyAI-JITAI app’s acceptability and clinical usability. Adults without a clinical anxiety or depressive disorder will be randomized to the 4-week JITAI or waitlist control group. The CBT-based app features a seven-day learning phase with Ecological Momentary Assessment (EMA) and optional geo-tracking to identify optimal intervention times and locations, followed by AI-driven JITAIs using reinforcement learning. Users will receive optional motivational support from a self-chosen buddy with shared app access. Feasibility will be evaluated using a formal framework. Assessments will be conducted at four time points: screening, pre-intervention (prior to randomization), mid-intervention (10 days following randomization), and post-intervention (4 weeks following randomization), complemented by qualitative interviews on user perspectives of the DyAI-JITAI.

**Discussion:**

This study aims to participatorily develop and assess the feasibility of a DyAI-JITAI app that bridges the intention-behavior gap by supporting CBT skill use in daily life and offering optional buddy-based motivation. The goal is to tailor the app to users’ needs and inform the design, procedures, and safety management of a future large-scale efficacy RCT.

## 1 Introduction

The global burden of major depressive disorders (MDD) has increased substantially over the past three decades. Between 1990 and 2021, the number of prevalent cases and disability-adjusted life years (DALYs) attributable to depressive disorders increased more than 1.8-fold worldwide. The global age-standardized prevalence rate and age-standardized DALY rate experienced marked increases from 2019 to 2021, rising by approximately 11% and 13%, respectively (1). Comorbid anxiety disorders are common in patients with MDD, affecting about 24–74% of MDD patients (2). In fact, people with depression are, on average, about six times more likely to also have an anxiety disorder compared to those without depression (3). Anxiety and depressive disorders not only diminish individual quality of life but also impose a substantial burden on society, including increased use of healthcare services, reduced work productivity, and early retirement (4). Preventive approaches are necessary to achieve early behavioral changes and to prevent the development of manifest disorders (5).

Psychological interventions have demonstrated effectiveness in reducing depressive and anxiety symptom severity in subthreshold depression with small to moderate effects (6) and lowering the incidence of depression (5, 7). These interventions, primarily based on cognitive-behavioral therapy (CBT), incorporate key components such as self-monitoring, behavioral activation, problem-solving, cognitive restructuring, assertiveness training, and relaxation techniques.

Given the limitations of face-to-face approaches, such as restricted accessibility, there has been growing interest in delivering preventive approaches through digital platforms. Evidence for digital interventions reveals both substantial promise and important limitations. Internet- and mobile-based interventions (IMIs) can reduce depressive and anxiety symptom severity (6, 8, 9) and the relative incidence of MDD in adults by 28% within a year (10), while mental health apps demonstrate overall small but significant effects on symptoms of depression and anxiety (11, 12). Research indicates that specific app features, such as CBT components, mood monitoring, and chatbot technology, are associated with larger effect sizes (12). In addition, substantial empirical evidence now supports CBT-based digital interventions as effective low-intensity interventions for subclinical symptom management and as resource-efficient, accessible tools for targeted prevention programs (13). However, similar to face-to-face approaches, preventive digital interventions do not benefit all users equally, and a notable proportion of participants still develop full-blown disorders despite intervention (10). When interventions fail to meet individual needs, this can result in high dropout rates, low adherence and engagement, and reduced effectiveness (14, 15)—an issue particularly pronounced in mental health related mobile interventions (16).

Most programs still follow a generic ‘one-size-fits-all’ approach, with little tailoring to individual needs. Although anxiety and depressive disorders often co-occur and share underlying mechanisms, prevention programs typically address them separately, leaving symptoms unaddressed. Developments in CBT have shifted toward transdiagnostic frameworks that span multiple disorder categories (17). These approaches target common underlying mechanisms, such as emotional avoidance, that contribute to various conditions, rather than focusing on disorder-specific symptoms like worry in anxiety disorder or worthlessness in depression. By addressing shared pathological processes, transdiagnostic interventions may more effectively treat comorbid presentations and subsyndromal symptoms that fall outside traditional diagnostic boundaries than disorder-specific approaches, potentially enhancing patient acceptability and treatment outcomes (18). Meta-analytic evidence suggests that both face-to-face as well as internet-based transdiagnostic CBT-based interventions are effective in treating depressive and anxiety disorders (19–21).

In addition, a major challenge is translating intentions and knowledge gained during interventions into concrete, health-promoting actions (22). A more dynamic and individualized approach in IMIs—delivering tailored support exactly when needed—has the potential to better support individuals in their daily lives according to their specific needs and to foster sustainable behavioral change (23). Adaptive digital interventions, like Just-In-Time-Adaptive-Interventions (JITAIs) and Ecological Momentary Interventions (EMIs), aim to achieve this level of individualization by levering new technologies like smartphones and sensors. Due to their similarities in design, the terms JITAIs and EMIs are often used interchangeably. However, JITAIs place greater emphasis on adaptation over time (24–26). Their goal is to tailor support to an individual’s changing internal and contextual state (27).

JITAIs are closely linked to Ecological Momentary Assessments (EMA) and smart sensing (28), which are commonly used to capture dynamic changes in individual variables such as mood, stress responses, and other psychological parameters, allowing for continuous, context-sensitive real-time monitoring. This high-frequency data collection provides detailed insights into daily mental health fluctuations and allows for dynamic adjustments of interventions based on current needs (29). EMAs can be active, involving brief self-reports multiple times per day (30), or passive sensing, capturing behavioral data (e.g., steps, geolocation) via built-in or external sensors on smartphones or smartwatches (31, 32). Moshe et al. (33) propose integrating active and passive data collection to predict internal states associated with depression or anxiety, thereby enabling JITAIs through optimized timing, format, and content of support. This aligns with the concept of smart sensing (also referred to as digital phenotyping or mobile sensing), which goes beyond passive digital marker collection by enabling data-driven predictions of individual outcomes and adaptive tailoring of interventions (34, 35).

To determine the appropriate timing of support, JITAIs continuously assess both a user’s vulnerability or opportunity state and their momentary receptivity using active and passive sensing data (27, 36). Vulnerability or opportunity refers to periods when an individual is either particularly susceptible to negative health outcomes or especially open to make positive behavior changes. Receptivity, in contrast, reflects a person’s current capacity and willingness to engage with supportive input (23, 27).

Although JITAIs in mental health contexts are still emerging, they represent a promising technological framework that may significantly advance the precision and effectiveness of mental health interventions (26, 37). Meta-analytic results on JITAIs targeting various behaviors of interest, such as healthy diet, mental health, addiction, and weight loss, showed moderate to large effect sizes for JITAI compared to waitlist-control conditions (*k* = 9, *g* = 1.65) and compared to non-JITAI (*k* = 21, *g* = 0.89) (38). EMIs have likewise demonstrated beneficial effects on mental health and well-being outcomes (39). A recent pilot randomized controlled trial evaluated the feasibility, acceptability, and preliminary efficacy of a self-guided, personalized, transdiagnostic, and mechanistic smartphone app targeting repetitive negative thinking in young people with depression and anxiety (40). The app is based on the JITAI model and incorporates cognitive behavioral therapy activities. It uses an algorithm that tailors a recommendation for an intervention designed to disrupt repetitive negative thinking based on responses to a brief EMA check-in that captures the levels of repetitive negative thinking, mood, context, and location. Acceptability was evidenced by sustained app engagement, with 90% of participants (26/29) actively using the application during week three, and 59% (17/29) continuing usage through week six. The trial demonstrated significantly greater reductions in depression, anxiety, and repetitive negative thinking among participants using the app compared to an inactive control group, with moderate to large effect sizes. These findings suggest potential efficacy for smartphone-based JITAIs incorporating CBT techniques to target specific mechanisms underlying depression and anxiety in youth populations. Another pilot study showed that a JITAI may reduce negative rumination by delivering rumination-focused-CBT interventions immediately following rumination episodes, thereby preventing subsequent rumination cycles triggered by the same stimuli (41). EMA was used to decide which interventions to deliver and when. These findings demonstrated the potential of JITAIs based on rumination-focused-CBT for reducing depressive symptoms. However, a systematic literature review revealed that current depression apps fail to implement true JITAIs (37). While 71% (20/28) of reviewed apps utilized self-reported outcomes and 29% (8/28) incorporated passive smartphone measurements, none leveraged these data to dynamically tailor intervention content and timing based on individual vulnerability or receptivity states.

In addition, while the use of JITAIs may improve individual responsiveness, they do not address the absence of interpersonal factors like social support, an important driver of engagement (42). Evidence suggests that integrating peers or family members into digital mental health interventions is feasible, acceptable (43, 44), and may enhance retention rates (45). This is particularly relevant given that user engagement remains a significant challenge in mental health apps (46), raising concerns about clinical utility and real-world transferability despite positive outcomes in controlled environments (12, 47). Combining JITAI mechanisms with dyadic social support components in preventive mental health applications may address these limitations by fostering supportive accountability and helping bridge the intention-behavior gap while remaining scalable and independent of professional resources, provided participant safety is maintained (48). Overall, although preliminary studies demonstrate promising feasibility, acceptability, and efficacy of JITAIs for treating depression and anxiety, further research is needed to evaluate the full potential of this integrated approach, particularly for preventing common mental disorders.

## 2 Aims

This study aims to develop and test a dyadic, AI-driven Just-In-Time Adaptive Intervention app (DyAI-JITAI) for the prevention of anxiety and depressive disorders in a non-clinical adult population. It comprises two consecutive stages. In stage I, a participatory design approach was used to co-develop the app with potential users. Through iterative qualitative methods, we identified and adapted intervention content and features based on expectations, experiences, needs, and preferences of the target group. Stage II involves a two-arm randomized controlled proof-of-concept study to assess the feasibility, acceptability, and preliminary clinical relevance of the DyAI-JITAI and the feasibility of a subsequent randomized controlled clinical trial. Specifically, we will evaluate (1) recruitment and sample characteristics, (2) data collection and outcome measures, (3) acceptability and satisfaction, (4) resource requirements, (5) user engagement and preliminary efficacy, and (6) potential risks and adverse effects. We hypothesize that the DyAI-JITAI app will be usable, acceptable, and effective in supporting CBT skill acquisition and behavioral change in daily life.

## 3 Methods

### 3.1 Stage I: participatory app development

#### 3.1.1 Study design

The intervention was developed using user-centered design principles (49) through collaboration between a multidisciplinary team comprising clinical researchers, public health specialists, technology developers, and design experts, in consultation with the target population (e.g., individuals with lived experiences). We collected qualitative data through a two-step participatory app development process. Three initial online focus groups informed content development for anxiety and depression prevention, while two follow-up groups evaluated design, usability, and practical application. Semi-structured interviews examined engagement factors, user attitudes, and needs. In usability tests, thinking-aloud method (50) with research assistants provided real-time feedback on the beta version. Trained interviewers conducted all 90-min sessions, which were recorded and transcribed verbatim. We analyzed data iteratively to rapidly refine prototypes, with final quality checks addressing technical issues. All procedures complied with General Data Protection Regulation (GDPR) requirements.

Individuals with lived experiences were eligible if they: (1) were aged 18 years or older, (2) had sufficient German language skills (native or self-reported C2 level), (3) had access to an internet-enabled smartphone, and (4) provided digital written informed consent and completed the screening. Exclusion criteria were (1) clinically relevant symptoms of anxiety or depression [Patient Health Questionnaire 9 (PHQ-9) ≥ 10 (51) and Generalized Anxiety Disorder Scale 7 (GAD-7) ≥ 10 (52)], (2) current or recent (past 6 months) treatment for anxiety or depressive disorders (self-reported), (3) indicate acute suicidal thoughts or behaviors (PHQ-9 Item 9 > 0).

We aimed to recruit 20 participants, based on recommendations for achieving data saturation (53). Final sample size was determined upon reaching data saturation, with *N* = 19 participants in the focus groups, *N* = 4 participants in the thinking-aloud tests and *n* = 5 in the qualitative interviews. Following guidelines for online focus groups (54), each group consisted of 4–5 participants.

Individuals were recruited via the study website, university mailing lists, social media (e.g., Instagram, Facebook), flyers, forums, and mental health websites. Interested individuals contacted the study team via the study website or email and received detailed study information, a data protection statement, and an informed consent form. Upon providing consent and contact details, they completed an online screening (SoSci Survey). Eligible individuals were then invited to schedule a qualitative assessment.

Qualitative data were analyzed using content analysis (55) combining inductive and deductive thematic approaches using MAXQDA-24. Two independent raters coded transcripts to identify themes and subcategories, with inter-coder reliability ensured through iterative consensus building following established guidelines (55). An online validation questionnaire was subsequently developed based on the analysis results and presented to interview participants to assess their agreement with findings.

The study was approved by the local Ethics Committee of the University of Marburg (CaYou2024-50k) and reported in accordance with the Consolidated Criteria for Reporting Qualitative Studies (COREQ) checklist (56). The trial protocol was developed following the updated SPIRIT guidelines (57). This study is registered on the Open Science Framework: https://doi.org/10.17605/OSF.IO/NSPKU.

#### 3.1.2 App content

The application’s infrastructure was provided by Pathmate Technologies, utilizing their Pathmate Cloud Platform (PMCP), which has evolved from the open-source intervention platform MobileCoach (58). Accordingly, the content was developed in alignment with the structural and functional specification of the platform. This includes a mobile chat-based intervention interface with predefined response options and the use of infocards for psychoeducational content. Additional features were developed to specifically align with our study design, such as the dyadic component (possibility to link activity in the app to a peer, friend or family member), badges (reward icons that can be earned for completing exercises to enhance motivation), and the integration of reminders to engage (push notifications), consistent with common recommendations for mental health apps (59). The app name was also chosen through a participatory process and resulted in CaYou (Care for you).

To deliver interventions at moments of highest receptivity, an algorithm was implemented on the PMCP platform following the concept of JITAIs (27). The algorithm uses tailoring variables such as individual sleep times, favorite exercises, and behavior-based patterns (e.g., times when users previously completed exercises). If GPS access is granted, the app identifies “favorable” locations based on past activity. Decision points occur several times per day and are based on user activities and contextual data. Interventions are delivered when conditions suggest high receptivity (e.g., at familiar times or places). The algorithm continuously adapts to user behavior.

The app content was developed based on the results of the focus groups and by reviewing scientific and clinical literature on CBT, including established CBT manuals for anxiety and depressive disorders as well as a transdiagnostic IMI manual for the prevention of anxiety and depression (60, 61). The intervention content includes transdiagnostic elements like psychoeducation, goal-setting, behavioral activation, cognitive restructuring, problem-solving, exposure, skills training, and relapse prevention. Further, we integrated thematic areas relevant to the prevention of depression and anxiety disorder and general mental health promotion such as: physical exercise (62), sleep (63), self-esteem (64) or mindfulness (65). A full list of the app’s content is listed in Table 1.

**Table 1:** App content.

Modules	Content
Basic modules	
Introduction	Overview of app content and technical instructions
Thoughts, feelings, and behavior	The interconnection of cognitions, feeling and behavior
Motivation, goals, and values	Identification of current challenges, personal goals and strengthening motivation for behavioral change
Thematic modules	
Emotion regulation	Identification and labeling of emotions, underlying needs, physiological responses, regulation and acceptance of emotions
Behavior activation	Interconnection of behavior and mood, depressive spirals, rewards, how to establish (versatile) activities
Adaptive thoughts	Facilitating and hindering cognitions, ABC-model, core beliefs and how to change them, cognitive biases
Anxieties and challenges	Avoidance and safety behaviors, exposure exercises
Problem-solving	Discrimination between solvable and unsolvable problems, 6-steps-plan
Relaxation	Relaxation techniques, their physiological and psychological effects, importance of consistent practice
Self-esteem	Self-esteem identification, core components, and internal dialogues, inner critic, benevolent companion, resources
Social skills	Social competencies, self-confidence, communication
Acceptance	Concept of acceptance, its therapeutic utility and practical application
Mindfulness	Concept of mindfulness, “what” (observe, describe, participate) and “how” (non-judgmentally, one-mindfully, effectively) skills
Self-care	Concept of self-care and self-compassion, positive affirmation, healthy habits
Physical exercise	Interconnection of physical activity and mental wellbeing, exploring dimensions of activity and exercises
Sleep	Psychoeducation and exercises on sleep phases, sleep disorders, and sleep hygiene
Confrontation with goals	SMART-goals, personal values
Sexuality and relationship	Psychoeducation on sexuality and sexual dysfunctions, sexual communication strategies communication about sexuality and intimacy
Closing module	Relapse prevention, review and outlook

#### 3.1.3 Qualitative results

Key themes from the focus groups included skepticism about the dyadic approach, concerns about GPS-based sensor tracking and data security, and feedback on multimedia content, interactive design, and support services. In the thinking-aloud sessions, participants emphasized the link between exercise and psychoeducation, progress tracking, voice-based input, and the burden of EMA questionnaires. Adaptations to the final app version were guided by technical feasibility, potential impact, and the current literature. Participant-driven changes included optional use of the dyadic feature and GPS tracking, more audio/video content, customizable design (e.g., coach icon), a clearly marked section for support services, improved integration of psychoeducation and exercises, and a reduced frequency of daily questionnaires from five to three (with five retained during the initial learning phase).

### 3.2 Stage II: Feasibility study

#### 3.2.1 Study design

**Figure 1:**
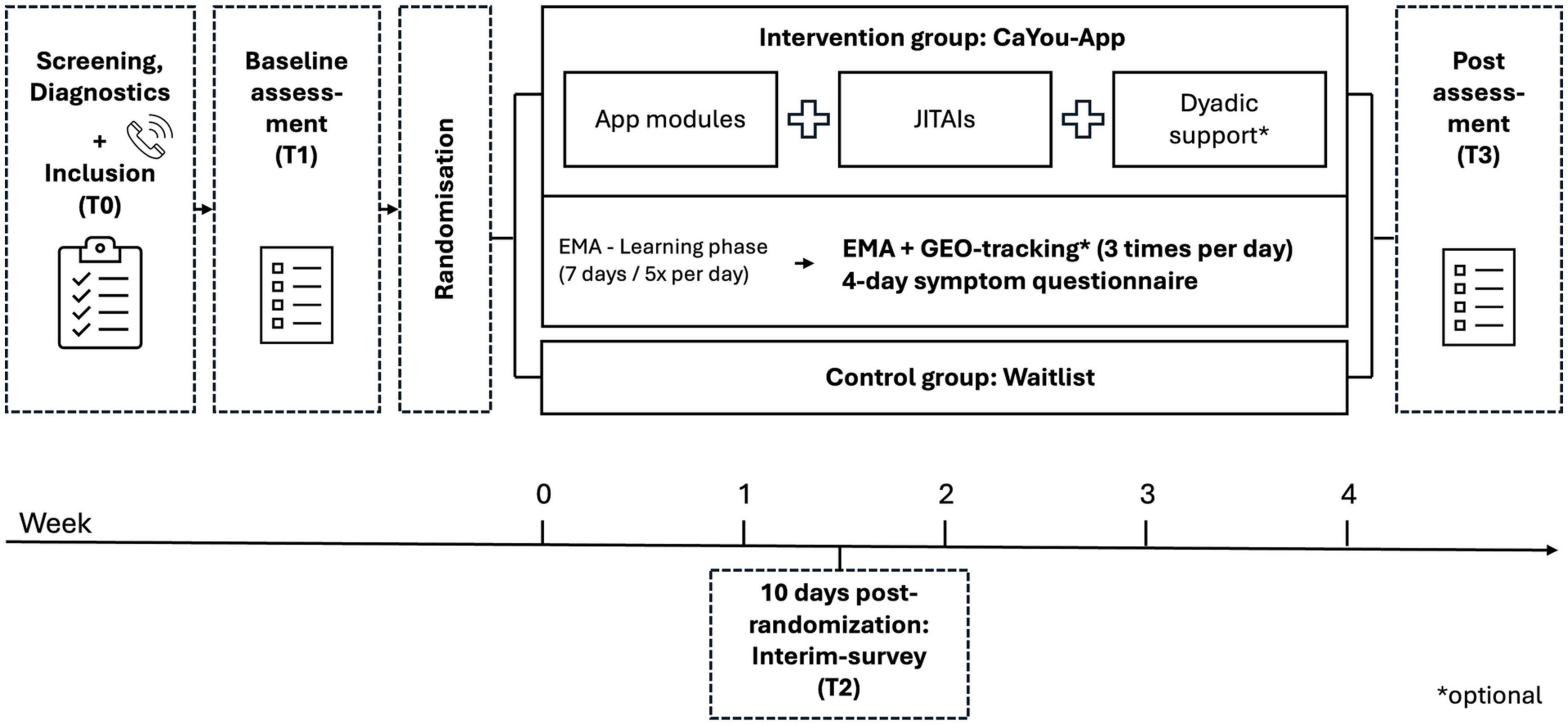
Study design. T0, Screening; T1, Baseline; T2, 10 days post-randomization; T3, 4 weeks post-randomization; JITAI, Just-in-Time Adaptive Interventions; EMA, Ecological Momentary Assessment.

In Stage II, a two-arm randomized-controlled proof-of-concept trial will compare the intervention group (IG) using the CaYou app for 4 weeks with a waitlist control group (WLC), who will receive access after 4 weeks. An overview of the Stage II design is shown in Figure 1.

The trial was approved by the local Ethics Committee of the University of Marburg (CaYou2025-05v) and will be reported in accordance with the Consolidated Standards of Reporting Trials (CONSORT) 2025 Statement and its extensions for pragmatic trials and psychological intervention trials (66). Qualitative analyses will follow the Consolidated Criteria for Reporting Qualitative Studies (COREQ) checklist (56). The trial protocol was developed according to the updated SPIRIT guidelines (57) and is registered on the Open Science Framework: https://doi.org/10.17605/OSF.IO/EYXD2.

##### 3.2.1.1 Recruitment and screening procedure

Participants will be recruited via the study website, university mailing lists, social media (e.g., Instagram, Facebook), flyers, forums, and mental health websites. Interested individuals can contact the study team via the study website or email and will receive written study information, a data protection statement, and an informed consent form. After signing the screening consent and providing contact details (name and email address), they will complete an online screening (SoSci Survey). Eligible individuals will be invited to a diagnostic telephone interview to confirm eligibility. Those who qualify will then provide full informed consent and complete the online baseline assessment. Participants are informed at the beginning that they will not receive monetary compensation for their participation in the study.

##### 3.2.1.2 Inclusion and exclusion criteria

Eligible participants must: (1) be 18 years or older, (2) have sufficient German language skills (native or self-reported C2 level), (3) have access to an internet-enabled smartphone, and (4) provide digital written informed consent and complete the baseline assessment. Exclusion criteria include (1) clinically relevant anxiety or depression symptoms (PHQ-9 ≥ 10 and GAD-7 ≥ 10), (2) a current or recent (past 6 months) diagnosis of an anxiety or depressive disorder [as assessed by the Structured Clinical Interview for DSM-5, SCID-5; (67)], (3) indication of acute suicidal thoughts or behaviors (PHQ-9 Item 9 > 0), (4) current psychotherapy or placement on a waiting list, and 5) self-reported psychotic disorders, or bipolar disorder (SCID-5).

##### 3.2.1.3 Randomization

Eligible individuals will be randomly allocated to the IG or WLC by an independent researcher using block randomization (blocks of 4 and 6 to minimize predictability in allocation) via Sealed Envelope.^1^ Participants will be informed of their group allocation; those in the IG will receive immediate access to the CaYou app, while WLC participants will gain access after 4 weeks.

#### 3.2.2 CaYou app

The CaYou app starts with a mandatory introductory module explaining its purpose, features, and content. This is followed by a 7-day EMA-learning phase, during which users receive five daily push notifications at random times. An AI-based reinforcement learning algorithm uses participants’ responses to notifications to identify optimal delivery times and locations. When participants confirm a prompt as well-timed, both the timestamp and GPS location, if allowed, are recorded. From week two to four, the system switch to a data-driven mode, delivering three EMAs per day based on previously identified optimal moments. Within a 4-h window between wake-up and bedtime, the algorithm monitors for suitable times or locations to trigger EMAs. If needed, prompts are delivered even without detected suitability to ensure appropriate spacing (minimum 4 h apart). Time and location feedback continues to refine the model throughout this phase. Following the fourth week, the algorithm is deactivated, and participants complete a final survey. While the AI individualize timing, the content is tailored to users’ mental well-being as assessed via daily EMA. The EMA includes the PHQ-4 (68) to monitor anxiety and depressive symptoms. If elevated symptoms are detected (PHQ-4 subscale score > 2), users receive targeted JITAI suggestions. Otherwise, they can select a topic of interest. JITAI exercises previously rated as helpful are saved as “favorites” and shown more frequently. Each JITAI is linked to an optional psychoeducative module.

The app content comprises one introductory module, two additional core modules to be completed at the start, and 15 optional topic-specific psychoeducative modules, each accompanied by a matching infocard that provides a concise overview. A final closing module focuses on reflection and relapse prevention after the 4-week intervention phase. All modules are delivered via an interactive chatbot that incorporates videos, audios, illustrations, fictional case examples from three personas illustrating challenges and solutions through speech bubbles, and interactive elements such as free-text and multiple-choice inputs. In addition, the app offers 67 JITAI exercises lasting between 2 and 15 min. These exercises are text- or audio-based, target specific goals (e.g., improving problem-solving skills), and provide practical, real-life strategies. Push notifications will be used daily to prompt users to complete EMA assessments and exercises, although all content is also freely accessible via a library within the app.

A central feature of CaYou is its dyadic component, which allows users to connect with a “Buddy”—such as a close friend or family member—who can provide support and motivation. Buddies can be linked through the app and view shared milestones like completed exercises, modules, or badges, thereby fostering interpersonal accountability and sustained engagement. Buddies do not access the app directly but receive automated email notifications when participants share achievements via standardized templates. A guide for the buddy will be created with study information and response templates for adherence alerts and participant achievements, designed to minimize mentor psychological burden while ensuring appropriate supportive communication.

#### 3.2.3 Sample size and power calculation

For stage II, the sample size was calculated following pilot study guidelines (69). To detect a very small effect (*d* < 0.3) on the PHQ-4 at post-assessment (*α* = 0.05, *β* = 80%), and accounting for an anticipated 20% dropout rate, *n* = 30 participants per group are required, yielding a total sample size of *N* = 60 participants.

#### 3.2.4 Assessments

##### 3.2.4.1 Data collection time points

Assessments will take place at screening (T0), baseline (T1), 10 days post-randomization (T2, only IG), and 4 weeks post-randomization (T3, post-intervention). Data collection includes online self-reports and telephone-based diagnostics (SCID-5). In-app assessments include daily EMA (PHQ-4) and repeated measures every 4 days (PHQ-4, TYDQ, CBTSQ [Things You Do Questionnaire and Cognitive-behavioral Therapy Skills Questionnaire]). In the IG, additional qualitative interviews after the app usage phase take place to explore their experiences with the study process, app usage, its acceptance, as well as facilitators and barriers to its application. Those who have discontinued the intervention will also be invited to identify barriers and reasons for dropout. An overview of all assessments and instruments is provided in Table 2.

**Table 2:** Assessment overview.

Instrument	Variable	Time of measurement					
		Screening	T0 Baseline	T1 10 Days	T2 Post	Daily	Course 4-days
Self-developed items	Inclusion criteria	x					
PHQ-ADS	Anxiety and depression	x					
BDI-II Item 9	Suicidal thoughts and behaviors	x			x		
PHQ-4	Anxiety and depression		x		x	x	x
Self-developed items	Sociodemographic data		x				
Self-developed items	Current medication		x		x		
ZUF-8	Treatment satisfaction			x	x		
Self-developed items				x	x		
TYDQ	Behaviors that promote mental health		x		x		x
INEP-On	Positive and negative effects of app use				x		
CBTSQ	CBT-Skills		x		x		x
SUS	Usability			x	x		
Self-developed items	Acceptability of data collection procedures				x		
Self-developed items	Concurrent treatment				x		
Self-developed items	Reasons for drop-out			x	x		
Diagnostic interviews							
SCID-5	Mental disorders, comorbidities, severity and chronicity		x				

PHQ-ADS, Patient Health Questionnaire Anxiety and Depression Scale; PHQ-4, Patient Health Questionnaire-4; BDI-2, Beck Depression Inventory-2; ZUF-8, Questionnaire for Patient Satisfaction 8; TYDQ, Things You Do Questionnaire; INEP-On, Inventory for Assessment of Negative Effects of Psychotherapy adapted to Online interventions; CBTSQ, Cognitive-Behavioral Therapy Skills Questionnaire; SUS, System Usability Scale; SCID-5, Structured Clinical Interview for DSM-5.

##### 3.2.4.2 Feasibility measures

A traffic light system with predefined criteria (see Table 3) was developed to assess feasibility. Following Orsmond and Cohn, six domains will be evaluated: (1) recruitment capability and resulting sample characteristics, (2) data collection procedures and outcome measures, (3) acceptability and suitability of intervention and study procedures, (4) resources and ability to manage and implement the study and intervention, (5) participant responses to the intervention, and (6) risks and adverse events (70).

1) Recruitment capability and resulting sample characteristics: Recruitment capability will be assessed by the number of individuals expressing interest in study participation, the intervention uptake rate, and the time required to reach the target sample. The traffic light system will evaluate whether the planned sample size (*N* = 60) is achieved within the 12-week recruitment period (see Table 3). Sample characteristics will be collected through sociodemographic data (e.g., age, gender, education, employment, relationship and parental status, residence size, nationality, ethnicity, migration status), family history of anxiety or depression, prior diagnoses, psychotherapy history, current medication or treatment, and experience with digital mental health tools.
2) Data collection procedures and outcome measures: The appropriateness of data collection will be assessed through EMA and T2 data completeness, psychometric evaluation of outcome measures, and feedback from the study team. Participant perspectives on acceptability and appropriateness will be gathered at T2 using self-developed items and in the qualitative telephone interviews based on a custom interview guide (IG participants only).
3) Acceptability and suitability of intervention and study procedures: Acceptability, satisfaction, and usability of the app will be measured using the German version (ZUF-8; *α* = 0.90, 8 items, score range: 8–32) of the Client Satisfaction Questionnaire (CSQ) (71), the System Usability Scale (SUS; *α* = 0.91, 10 items, score range: 0–40) (72), and self-developed items. At T2, qualitative telephone interviews will further explore participants’ experiences with the app’s features, content, and overall impressions. Intervention adherence will be measured via user engagement metrics (e.g., number of modules and exercises completed, usage days during the 4-week period, buddy interactions, and optional sensor data). Reasons for dropout will be assessed through self-developed items and within the qualitative interview at T2.
4) Resources and ability to manage and implement the study and intervention: The study team will document time and personnel resources needed for app development, study administration, and participant support.
5) Participant responses to the intervention: Symptoms of anxiety and depression will be measured using the Patient Health Questionnaire Anxiety and Depression Scale (PHQ-ADS; *α* = 0.88–0.92, 16 items; score range: 0–48) (73), which combines the Patient Health Questionnaire 9 (PHQ-9; *α* = 0.89, 9 items, score range: 0–27) (51, 74) and the Generalized Anxiety Disorder Scale 7 (GAD-7; *α* = 0.89, 7 items, score range: 0–21) (75). The PHQ-ADS has shown high internal consistency (*α* = 0.88–0.92). Cognitive behavioral skills will be measured using the Cognitive-behavioral Therapy Skills Questionnaire (CBTSQ; *α* = 0.90, 16 items, score range 16–80), covering behavioral activation (*α* = 0.85) and cognitive restructuring (*α* = 0.88) (76). The Things You Do Questionnaire (TYDQ; *α* = 0.90, 15 items, score range: 0–60) (77) will assess engagement in positive routines and meaningful activities, with higher scores indicating greater engagement (*α* = 0.90). Qualitative interviews at T2 will explore the perceived effects of the app from the IG participants’ perspective.
6) Risks and negative events: The benefit–risk ratio will be assessed by tracking adverse events, including reliable symptom improvement and deterioration according to the reliable change index (RCI) by Jacobson and Truax for the PHQ-ADS, indicating a decrease or increase of >1.96 from T1 to T3 as well as suicidal thoughts and behaviors (78). These will be measured at T2 using the INEP-On (Inventory for Assessment of Negative Effects of Psychotherapy adapted to online interventions; *α* = 0.86, 21 items) (79), item 9 of the PHQ-ADS (73), and item 9 from the BDI-II (80). Qualitative interviews will further explore perceived negative effects of the app.

**Table 3:** Traffic light system for assessing feasibility.

Traffic light system for assessing feasibility	Green	Yellow	Red
1. Participants recruited during the planned period (%)	*N* = 60 (100%)	*N* = 45–59 (75–98%)	*N* < 45 (<75%)
2. Participants with complete data at post-assessment (percentage of the total sample)	>70%	50–70%	<50%
3. Completed EMA data (at least one EMA completed per day; percentage of the total sample)	>60%	40–60%	<40%
4. Omega value for two-thirds of the outcome questionnaires	≥0.70	0.60–0.69	<0.59
5. Satisfaction (ZUF-8)	*M* > 26	*M* = 24–26	*M* < 24
6. Pre-post change in the PHQ-4 scores compared to the control group (depression and anxiety subscales)	Baseline score ≤3: No reliable deterioration, indeterminate, or reliable improvement Baseline score > 3: No deterioration or improvement reliable to deterioration and improvement	No improvement reliable to improvement in participants with a baseline score > 3	Symptom deterioration reliable to deterioration
7. Risk–benefit ratio	Positive	Negative: Isolated negative intervention-related events, that can be addressed by revising the intervention or study	Negative: Serious adverse intervention-related events (e.g., suicidal thoughts), symptom deterioration

EMA, Ecological Momentary Assessment; ZUF-8, Questionnaire for Patient Satisfaction 8.

##### 3.2.4.3 Qualitative interviews

At T2, qualitative interviews with IG participants will explore experiences related to feasibility criteria (see Table 3), including user satisfaction, usability, and practical applicability in daily life. To ensure data saturation, at least 20 participants will be interviewed (53). Interviews will be conducted by trained staff, audio-recorded, and transcribed verbatim using a standardized guide.

#### 3.2.5 Statistical analyses

##### 3.2.5.1 Quantitative data analysis

The intervention is considered feasible if all criteria in the traffic light system are rated yellow or green (see Table 3). Yellow ratings will prompt internal discussion and minor adjustments; red ratings indicate the need for major revisions, with input from external experts. Quantitative baseline data will be analyzed descriptively. Mental health outcomes will be examined using analysis of covariance (ANCOVA) to assess between-group pre-post changes, and time series analyses will explore individual symptom trajectories based on 4-day intervals.

##### 3.2.5.2 Qualitative data analysis

Qualitative interviews will be analyzed using a qualitative content analysis approach (55), combining inductive and deductive thematic analysis in an iterative process with MAXQDA-24. Transcripts will be coded by two independent raters to ensure inter-coder reliability, following established guidelines (55). The coding guide will be developed through iterative consensus. Based on the findings, an online validation questionnaire will be created and presented to interview participants to assess agreement with the results.

#### 3.2.6 Safety concept

Data on adverse and serious adverse events will be collected at different assessment points throughout stage II as recommended (81). Suicidal thoughts and behaviors will be assessed with PHQ-ADS item 9 (73) and BDI-II item 9 (80) at screening, and INEP-On item 20 at T2. Other negative events will be assessed with the INEP-On at T2. Relevant indications may also emerge from participants’ responses during the screening or qualitative post-intervention interview.

A detailed safety protocol, based on existing recommendations (77) outlines procedures for managing negative events. These include automated provision of help resources via questionnaires and email, and individual therapeutic phone consultations following a structured emergency plan tailored to the severity of suicidal ideation. A licensed psychotherapist will be available for consultations, and screening interviewers will be trained and supervised in implementing the emergency plan. All adverse events will be documented.

## 4 Discussion

This paper presents the study protocol and concept of one of the first feasibility and acceptability studies of a dyadic, AI-driven JITAI app for the prevention of anxiety and depressive disorders. Developed using a participatory research approach, the CaYou app integrates transdiagnostic CBT components. The study aims at providing early proof-of-concept regarding feasibility, acceptance, and preliminary efficacy of the app. Objective usage data and participant feedback will inform key areas for optimization and guide the design of a future full-scale randomized controlled effectiveness trial.

Despite the innovative approach, the proposed DyAI-JITAI app study also faces several challenges and risks during implementation. (1) A key challenge lies in identifying optimal moments for delivering interventions. The efficacy of the app may be influenced by how accurately the algorithm recognizes patterns and adapts to individual users. This could be more challenging if users have less consistent daily routines during the learning and intervention phases or if their engagement is low, for example due to intervention or assessment fatigue, or a general lack of interest (42). Although JITAIs are designed to adapt to users’ receptivity, reduce intervention fatigue, and support daily-life transfer, these issues may persist. (2) The decision points of the JITAI may not be optimally configured due to limited empirical evidence on the ideal frequency and timing—for instance, how long notifications should remain visible on smartphones, or how many exercises and prompts should be offered and displayed each day (26)—increasing the risk of missing critical intervention windows. (3) Another potential challenge is the buddy’s role in supporting behavior change. While social support can improve engagement, buddies may feel uncertain about how to respond effectively. As the support is intended to be one-sided, perceived asymmetries in responsibility or emotional reciprocity could strain the relationship and reduce the support’s impact. (4) Technical issues or system-generated recommendations that feel irrelevant to users may also lead to frustration and lower motivation for behavior change (42). (5) Recruitment may be difficult due to privacy or ethical concerns regarding active and passive EMA, especially among individuals with subclinical anxiety and depression (83). In addition, the abundance of freely available mental health apps, which are often not evidence-based (84), may reduce the appeal of participating in a study that requires more effort (6). The generalizability of the EMA and geolocation-based approach may be limited by several factors. Users in rural areas may experience challenges with inconsistent cellular coverage and GPS signal quality, affecting location-based triggers and real-time data collection. Additionally, the digital divide may create barriers for populations with limited smartphone access or digital literacy, including older adults and individuals with lower socioeconomic status.

## 5 Conclusion

The planned study will offer valuable insights into the real-world feasibility, acceptability, and preliminary clinical relevance of a dyadic, AI-driven JITAI app for the prevention of anxiety and depressive disorders. By integrating structured qualitative data, it will also deepen our understanding of how JITAIs may help close the intention-behavior gap—an essential factor in preventive mental health. The study follows a clear JITAI development framework, enhancing transparency and replicability while generating critical knowledge to guide future evidence-based JITAI designs. By delivering personalized, context-sensitive support in moments of needs, JITAIs can increase the relevance of interventions, thus having the potential to enhance engagement and adherence over time. This mechanism is crucial, as adherence and engagement are closely linked to efficacy and therefore important for behavior change (85). Ultimately, it lays important groundwork for developing effective, scalable interventions that promote sustainable behavior change in everyday life.

## Footnotes

^1^ https://www.sealedenvelope.com/
